# Computational Methods in the Design of Anticancer Drugs

**DOI:** 10.3390/ph17040404

**Published:** 2024-03-22

**Authors:** Marialuigia Fantacuzzi, Mariangela Agamennone

**Affiliations:** Department of Pharmacy, University “G. d’Annunzio” of Chieti-Pescara, Via Dei Vestini, 31, 66100 Chieti, Italy

In recent years, continuous progress has been made in the development of new anticancer drugs, and several compounds (small molecules, engineered antibodies, immunomodulators, etc.) have been approved, dramatically changing the landscape of tumor treatment [1]. Despite these efforts, cancer remains a major threat to human health and one of the leading causes of death worldwide [2]. This underscores the need for an even better understanding of the molecular mechanisms behind cancer initiation and progression. To date, the advent of immunotherapy, gene therapy and molecular targeted therapy has revolutionized the treatment of most cancers. In targeted therapy, the genetic signature of each type of cancer is targeted with drugs designed to act against actionable driver genes, avoiding the side effects of conventional chemotherapy and improving treatment efficacy. The advent of cancer immunotherapy and gene therapy has enriched the available armamentarium in the fight against this pathology, even with some limitations [3]. The efforts of the scientific community against cancer can also be seen in the number of drugs approved by the FDA for cancer treatment in 2023 (15 out of a total of 55 new drugs) [4].

Over the past few decades, computational methods have become an essential tool in the drug design process because they can reduce research costs and accelerate the development process [5]. Several factors have been contributing to the expansion of in silico applications. The increasing availability of 3D macromolecule structures through experimental (X-ray or cryo-EM) or computational methods (AlphaFold) [6] allows us to study most of the genome. The development of supercomputers enables the atom-based simulation of even larger systems. The availability of ultra-large compound libraries, which expand the explorable chemical space through screening campaigns, is another important factor. Furthermore, the growing application of artificial intelligence algorithms to drug discovery is having an increasing impact: AI algorithms are being applied in a variety of areas, such as the aforementioned protein structure prediction, QSAR/QSPR, structure-based modeling, and the prediction of AD-ME/toxicity profiles [7].

The application of computational methods in the design of anticancer drugs has proven to be very effective [8]. Given the wide variety of tumor types and the large number of possible pharmacological targets, this is a challenging area of research [2].

For the Special Issue on “Computational Methods in the Design of Anticancer Drugs”, we aimed to collect the most recent discoveries in the field of anticancer drug design using computational methods. The 11 articles (8 papers and 3 reviews) cover a wide range of topics, from pharmacophore modeling to molecular docking, molecular dynamics, and ADMET prediction, and focus on many different targets, highlighting the diverse target landscape in cancer treatment.

Bülbül et al. (contribution 1) focused on the development of novel selective HDAC3 (Histon DeACetylase 3) inhibitors containing the alkylhydrazide zinc-binding group. They generated and evaluated pharmacophore and atom-based QSAR models, and the binding mode of compounds was determined using molecular docking and molecular dynamics simulations. The developed models provide a clear explanation for the in vitro data.

Moreover, Córdova-Bahena et al. (contribution 2) generated a pharmacophore model using a set of well-known Casein Kinase 1 isoform epsilon (CK1ε) inhibitors. The resulting model was used to screen a library of FDA-approved drugs for repositioning purposes. Molecular docking and molecular dynamics were used to analyze new compounds. The antineoplastic drug Etravirine, which activates the WNT pathway in osteosarcoma cells by increasing the expression of the cyclin-dependent kinase (CDK) inhibitor p21, emerged as a CK1ε inhibitor. 

In addition, Bhujbal et al. (contribution 3) focused on Polo-like kinase 1 (PLK1) inhibitors, which can be used to treat various types of cancer, such as lung, colon, prostate, ovarian, breast, melanoma, and AML. They performed hybrid 3D-QSAR and molecular docking to design potent and selective inhibitors. Two compounds showed good IC_50_ values.

Crocetti and coworkers (contribution 4) used a ligand-based technique to develop more potent fatty acid binding protein 4 (FABP4) inhibitors, starting with a known pyrimidine ligand and applying bioisosteric replacements and scaffold hopping in the pyrimidine skeleton. They synthesized and biologically tested novel 4-amino and 4-ureido-pyridazinone-based compounds as FABP4 inhibitors. The molecular docking study confirmed the ability of the most active molecules to better interact inside the FABP4 binding pocket.

Al-Zahrani et al. (contribution 5) virtually screened 1289 flavonoids using molecular docking to the mitogen-activated protein kinase (MAPK) MEK1. ADMET prediction and 100 ns molecular dynamics (MD) simulations were then applied to the top five docked compounds, revealing them as promising potent inhibitors. 

Franco et al. (contribution 6) focused on the inhibition of nicotinic acid phospho-ribosyl transferase (NAPRT), the rate-limiting enzyme of the Preiss–Handler NAD biosynthetic pathway, which can overcome resistance to nicotinamide phosphoribosyl transferase (NAMPT) inhibition and lead to better anti-tumor effects. Selected hits from the virtual screening were tested in a cellular assay using the ovarian cell line OVCAR-5, and the recombinant hNAPRT and showed a synergistic effect with the NAMPT inhibitor FK866. 

The same research group (contribution 7) performed a structure-based virtual screening on a 537,009 drug-like compound library and identified two additional chemical scaffolds that functioned as NAPRT inhibitors. The new compounds showed comparable anti-cancer activity with respect to the previously discovered NAPRT inhibitor, 2-hydroxynicotinic acid (2-HNA), a better predicted solubility, and favorable drug-like properties.

Bartelink et al. (contribution 8) applied a computational method to develop a physiological pharmacokinetic (PBPK) model to predict the image quality (tumor-to-lung contrast) of three PET radiotracers binding the epidermal growth factor receptor tyrosine kinase (EGFR TKI PET/CT: ^11^C-erlotinib, ^18^F-afatinib and ^11^C-osimertinib), used to assess EGFR overexpression and mutation in NSCLC. The model was also developed to predict the uptake of healthy tissue in three radiolabeled EGFR ligands.

Finally, there are three reviews in this Special Issue. One, written by Wang et al. (contribution 9), focuses on applications of artificial intelligence in the design of anticancer drugs, demonstrating the basic ideas behind these techniques, as well as their advantages and disadvantages. The authors reviewed the literature from the past decade, focusing on all articles presenting computational studies using AI to assist in the identification of effective cancer treatments. In addition, the authors provided a compilation of useful databases (omics, chemical compounds, drugs, etc.) as a valuable tool in the application of AI for drug discovery.

Primavera et al. (contribution 10) focused on small-molecule AKT inhibitors that were validated for anticancer activity using computer-aided drug design methods. The authors provided an introductory analysis of AKT structural features and binding sites. Then, a comprehensive analysis of inhibitors identified via different approaches (pharmacophore screening, docking, QSAR, machine learning) is reported, distinguishing between orthosteric and allosteric binders.

In our review (contribution 11), we examined the most relevant papers that elucidated the binding mechanism of PD-L1 with PD-1 and small molecules through computational analyses. In particular, the hot spot residues involved in the interaction between PD-L1 and PD-1 and the PD-L1 dimerization induced by small molecule binding are described. Virtual screening campaigns, mainly structure-based, that were performed to identify new small-molecule PD-L1 binders are also reported. 

As Guest Editors, we hope that the findings included in this Special Issue will inspire further investigations in this challenging field.
